# Thyroid cytology in pediatric patients: a single-center study from 2015 to 2023—is there a necessity for distinct treatment approaches for patients with and without autoimmune thyroiditis?

**DOI:** 10.1007/s00428-024-03959-6

**Published:** 2024-11-05

**Authors:** Monika Kujdowicz, Dominika Januś, Jan Radliński, Aleksandra Kiszka-Wiłkojć, Anna Taczanowska-Niemczuk, Damian Młynarski, Wojciech Górecki, Jerzy B. Starzyk, Dariusz Adamek

**Affiliations:** 1 Department of Pathomorphology, Jagiellonian University Medical College, Grzegórzecka 16 Street, 31-531 Krakow, Poland; 2 Department of Pathomorphology, University Children Hospital in Krakow, Krakow, Poland; 3 Department of Pediatric and Adolescent Endocrinology, Chair of Pediatrics, Institute of Pediatrics, Jagiellonian University Medical College, Krakow, Poland; 4 Department of Pediatric and Adolescent Endocrinology, University Children Hospital in Krakow, Krakow, Poland; 5 Student Scientific Group of Pediatric Endocrinology, Department of Pediatric and Adolescent Endocrinology, Jagiellonian University Medical College, Krakow, Poland; 6 Department of Pediatric Surgery, Institute of Pediatrics, Jagiellonian University Medical College, Krakow, Poland; 7 Department of Pediatric Surgery, University Children Hospital in Krakow, Krakow, Poland

**Keywords:** Thyroid cytology, Bethesda system, Pediatric thyroid nodules, Risk of malignancy, Autoimmune thyroiditis

## Abstract

The management of thyroid nodules is guided by the cytological classification provided by The Bethesda System for Reporting Thyroid Cytology. Notably, the biology of thyroid tumors in pediatric patients differs from that in adults, and there is limited research focused on pediatric cases. This study aimed to assess the effectiveness of the Bethesda system in pediatric patients treated at the largest tertiary pediatric thyroid center in Poland between 2015 and 2023. A retrospective analysis was conducted on 566 patients with thyroid nodules, of whom 555 underwent fine-needle aspiration biopsy (FNAB). A total of 217 patients underwent thyroid surgery. Of these, 206 had previously undergone FNAB with cytological evaluation at our center, while 11 patients underwent thyroid surgery due to a RET mutation or the need for an extended procedure. The initial FNAB results showed distribution across Bethesda categories as follows: 7.6% for category I, 54.6% for category II, 20.9% for category III, 4.1% for category IV, 7.6% for category V, and 5.6% for category VI. Among patients who underwent surgery, the distribution of Bethesda categories I through VI was 2.9%, 25.2%, 29.1%, 8.3%, 19.4%, and 15%, respectively. The risk of malignancy (ROM) from the initial FNAB was estimated at 33.3%, 11.5%, 22.2%, 4.8%, 84.4%, and 96.8% for Bethesda categories I through VI, respectively. In patients with autoimmune thyroiditis (AIT), the ROM was higher than in non-AIT patients for Bethesda categories I through IV, while it was lower in category VI. The sensitivity for detecting non-benign neoplasms across Bethesda categories III through VI was approximately 86% in both AIT and non-AIT patients. However, for papillary thyroid carcinoma, sensitivity in Bethesda categories V and VI was 86% in non-AIT patients but decreased to 61.5% in AIT patients. These findings emphasize the importance of considering surgical intervention in pediatric patients with Bethesda III-VI cytology, particularly in those with AIT.

**Supplementary information:**

The online version contains supplementary material available at 10.1007/s00428-024-03959-6.

## Introduction

The diagnosis of thyroid dysfunction and goiter relies on both biochemical testing and ultrasonography (USG). When focal abnormalities, such as nodules or irregular lesions—especially those that are hypoechoic and enlarging—are detected on USG, fine needle aspiration biopsy (FNAB) is performed as a screening method for thyroid cancer [1, 2]. FNAB is also conducted prior to surgery in cases of large goiters causing compressive symptoms and before total thyroidectomy for Graves’ disease unresponsive to conservative treatment (e.g., thiamazole), or in patients with contraindications to radioactive iodine (^131^I) therapy, such as young age.

Thyroid pathology in children differs significantly from that in adults [1–5]. In pediatric patients, the differential diagnosis of thyroid lesions includes developmental anomalies, such as thyroglossal duct cysts, midline neck cysts, and the presence of intrathyroidal ectopic thymus. The thyroid gland in children may also display a fine-vesicular structure, characteristic of fetal development. Additionally, pediatric patients tend to have a higher number of lymph nodes in surgical specimens compared to adults [1, 2]. Pediatric thyroid cancers differ in their molecular characteristics and clinical behavior [1–5]. Although papillary thyroid carcinoma (PTC) accounts for 90% of pediatric thyroid malignancies, similarly to adults, it shows a lower prevalence of the BRAF V600E mutation, a higher rate of metastasis, and a more balanced female-to-male ratio [3, 4].

In 2015, Poland adopted the Bethesda System for Reporting Thyroid Cytopathology (TBSRTC) as the standard for categorizing fine-needle aspiration cytology findings, in line with recommendations from the American Thyroid Association (ATA) [5]. Prior to this, the Papanicolaou classification system was used [6]. As of 2023, the TBSRTC consists of six categories: I, non-diagnostic (ND); II, benign (B); III, atypia of undetermined significance (AUS, previously including follicular lesion of undetermined significance [FLUS]); IV, follicular neoplasm (FN, previously including suspicion for follicular neoplasm [SFN]); V, suspicious for malignancy (SM); and VI, malignant (M) [7]. Evaluating differences in outcomes between various versions of the Bethesda System is complex [8]. The primary purpose of cytology as a screening tool is to guide decision-making regarding whether thyroid surgery is indicated and, if so, the extent of surgery required (lobectomy versus total thyroidectomy, with or without lymph node dissection).

Histological examination of resected thyroid tissue remains the gold standard for diagnosis. It provides critical information about the type of lesion, classified according to the World Health Organization (WHO) system, and the cancer stage, as defined by the American Joint Committee on Cancer (AJCC). This information is essential for determining the course of further treatment [9, 10]. According to the WHO classification, thyroid tumors are categorized into developmental anomalies, follicular cell-derived neoplasms (FCDNs), C-cell-derived neoplasms, salivary gland-type neoplasms, embryonal neoplasms, neoplasms with uncertain histogenesis, and thymic neoplasms. FCDNs are the most common and are subdivided into three groups: benign tumors (which include follicular nodular disease [FND], follicular adenoma, follicular adenoma with papillary architecture, and oncocytic adenoma [OA]), low-risk neoplasms (including non-invasive follicular thyroid neoplasm with papillary-like nuclear features [NIFTP], tumors of uncertain malignant potential [follicular and well-differentiated, FT-UMP and WDT-UMP], and hyalinizing trabecular tumor [HTT]), and malignant neoplasms (such as papillary thyroid carcinoma [PTC], invasive encapsulated follicular variant of PTC, follicular thyroid carcinoma [FTC], oncocytic thyroid carcinoma [OTC], high-grade follicular cell-derived carcinoma [FCDC], and anaplastic carcinoma [AC]) [9].

The most common thyroid malignancy, papillary thyroid carcinoma, as well as the rarer medullary carcinoma, are typically treated with total thyroidectomy. In contrast, other thyroid lesions are generally managed with lobectomy. In cases of malignant neoplasms, total thyroidectomy is followed by radioactive iodine (^131^I) therapy. However, benign tumors and low-risk neoplasms greater than 4 cm in diameter or causing compressive symptoms are routinely treated with lobectomy without subsequent radioactive iodine therapy [11, 12].

Autoimmune thyroiditis (AIT) encompasses a group of conditions in which the immune system generates antibodies against the thyroid gland, including anti-thyroperoxidase, anti-thyroglobulin, and anti-TSH receptor antibodies. The thyroid gland is also infiltrated by lymphocytes. AIT affects approximately 1.4–6% of children, with higher prevalence in adolescents, and onset has been observed in children as young as 1 year old [13–15]. The most common pediatric presentation is Hashimoto’s thyroiditis, which can manifest as hypothyroidism, euthyroid goiter, or, less frequently, as hashitoxicosis (hyperthyroidism). In rare cases, hashitoxicosis may transition to Graves’ disease with hyperthyroidism. Treatment for hypothyroidism involves levothyroxine, while hyperthyroidism is managed with antithyroid medications, radioactive iodine (^131^I), or surgery.

The etiology of AIT is multifactorial and is associated with genetic variants such as HLA-DR3, HLA-DR5, and CTLA-4. AIT is also linked to other autoimmune disorders, including celiac disease, type 1 diabetes mellitus, Addison’s disease, alopecia, and vitiligo [14, 16]. Additional risk factors for AIT include genetic syndromes (e.g., Turner syndrome, DiGeorge syndrome, trisomy 21), viral infections, obesity, iodine excess, and deficiencies in vitamin D and selenium. There is evidence suggesting that AIT increases the risk of papillary thyroid carcinoma (PTC) and follicular thyroid carcinoma (FTC) in thyroid nodules, although data on this association remains conflicting [13, 14]. Thyroid nodules are found in approximately 31.5–64% of pediatric AIT patients, compared to only 2% in non-AIT pediatric patients [1, 14, 17]. Pediatric thyroid nodules carry a 9.2–50% risk of malignancy, which is higher than in adults, where the risk ranges from 5 to 15% [1, 14, 17].

Although pediatric thyroid lesions differ from those in adults, thyroid cytology in children is assessed using the same Bethesda System applied to adult cases. The existing literature on malignancy risk in pediatric thyroid cases remains sparse, and the 2023 update of the Bethesda System references Heider et al.’s 2020 report as a key source of data [7, 18]. Here, we present findings from our experience with thyroid cytology in pediatric patients treated at the largest tertiary thyroid pediatric center in Poland between January 2015 and December 2023.

## Methods

This study was approved by the institutional review board of the Jagiellonian University Medical College (The Committee for Scientific Research Ethics; approval number: 118.0043.1.103.2024). Written informed consent for standard medical procedures was obtained from all participants and/or their parents/legal guardians.

### Subject

A retrospective analysis of medical records was conducted, focusing on thyroid FNAB cytology and histology data from the University Children’s Hospital’s Department of Pathology in Krakow. Data collected included patient age, gender, and the presence or absence of autoimmune thyroiditis (AIT) in their medical history. All patients were euthyroid before undergoing FNAB.

We retrieved thyroid FNAB and histopathology records from January 2015 to December 2023. Cytology was classified according to the Bethesda System, and histology was categorized following the World Health Organization’s tumor classification and the American Joint Committee on Cancer staging guidelines. Old pathology reports were re-evaluated and verified against updated nomenclature standards. Microphotographs of cytological samples were captured using an Olympus BX53 white-light microscope with an Olympus DP27 digital camera. Data were organized in Microsoft Excel (Office 365, Microsoft, Redmond, WA, USA), with each patient’s information consolidated into a single row to avoid data duplication. Patient information was pseudonymized, and unique identification numbers were assigned during this process.

### Statistics

The demographic characteristics were summarized using descriptive statistics. Categorical variables were expressed as percentages.

Nodule size was calculated based on the diameter of the largest biopsied nodule for each patient, even in cases of multiple follicular nodules. The average nodule size was then determined for the entire patient population.

Some patients underwent surgery following FNAB, and in certain instances, multiple FNABs were performed on the same patient. To assess the diagnostic accuracy of thyroid cytology, the first and last cytology results were compared with histological findings. The performance of FNAB in Bethesda categories was evaluated by calculating sensitivity, specificity, positive predictive value (PPV), negative predictive value (NPV), and overall accuracy using a contingency table. FNAB outcomes were compared to the “gold standard” of histopathological examination.

The risk of malignancy (ROM) between AIT and non-AIT groups (post-surgical patients) for each Bethesda category was analyzed using a test for two proportions with normal approximation. Depending on the sample size, either the Chi-squared test (for populations smaller than 40; n1 + n2 < 40) or Fisher’s exact test (n1 + n2 ≥ 40) was applied, using the OriginPro 2024 software (OriginLab Corporation, Northampton, MA, USA). A two-tailed *p*-value of < 0.05 was considered statistically significant.

## Results

### The cohort

A total of 566 patients underwent morphological examination of the thyroid. The female-to-male ratio was 390:176, and the average patient age was 13.4 years (SD ± 3.2, range 2–18 years). The ratio of non-AIT to AIT patients was 397:169.

Fine-needle aspiration biopsy (FNAB) was performed on 555 of these patients. Between 2015 and 2023, 684 biopsy procedures were conducted. In some cases, multiple sites were biopsied during a single procedure, resulting in a total of 861 biopsy sites. The number of aspirations per FNAB ranged from 1 to 3, depending on the nodule size and the volume of tissue aspirated. Larger or more fibrous nodules required more punctures to obtain adequate samples.

At our center, 217 patients with thyroid abnormalities underwent surgery between 2015 and 2023. Of these, 206 had surgical follow-up (SFU) after FNAB, and 11 had surgery without TBSRTC. Among 118 patients who underwent repeated biopsies, 41 had SFU. Ten of these patients were diagnosed with malignant neoplasms, including nine cases of papillary thyroid carcinoma (PTC) and one case of follicular thyroid carcinoma (FTC). Additionally, two patients were diagnosed with low-risk neoplasms: one case of non-invasive follicular thyroid neoplasm with papillary-like nuclear features (NIFTP) was reclassified from Bethesda category II to III, and one case of follicular tumor of uncertain malignant potential (FT-UMP) was reclassified from Bethesda category II to IV. In one patient, FTC was upgraded from Bethesda category II to III.

In the PTC cases, the initial and repeated FNAB categories were as follows: I and III, I and VI, II and II, II and III, three cases of II and V, and IV and V. In an interesting case involving repeated biopsies, the patient consistently received a Bethesda category III diagnosis. The repeated biopsies were prompted by the presence of psammoma bodies observed on ultrasound in the thyroid and lymph nodes. However, no thyrocytes were detected in the lymph nodes on cytological examination. Subsequent histological analysis revealed psammoma bodies in both the thyroid and lymph nodes, leading to a final diagnosis of PTC upon second pathology consultation.

For patients who had repeated FNABs without surgical follow-up (either due to planned surgery or benign FNAB results), the risk of malignancy (ROM) was not impacted. ROM as determined by the Bethesda System for Reporting Thyroid Cytopathology (TBSRTC) can be influenced by several factors, such as the patient’s condition and access to medical care.

Table 1 presents data on the number of FNABs, SFU cases, gender ratios, nodule sizes, general histological groups, and ROM according to the TBSRTC categories, regardless of whether patients had autoimmune thyroiditis (AIT). Of the initial 42 Bethesda category I cases, 15 were upgraded. Among the 303 cases of Bethesda category II, 12 were upgraded. One of the 116 patients initially categorized as III was upgraded to Bethesda category IV, with histological follow-up confirming FTC. None of the 23 categories Bethesda IV cases was upgraded. Of the 42 patients initially diagnosed as Bethesda category V, only one, after a pathology consultation, was reclassified as category VI. There were 31 patients initially diagnosed in the VI category. Eleven patients underwent surgery without prior FNAB due to a RET mutation or the need for extended surgery.

**Table 1:** The outcomes for the Bethesda System for Reporting Thyroid Cytopathology (TBSRTC) for all patients as a screening test in the first FNAB, the final FNAB before planned surgery, and surgical follow-up (SFU)

Category of TBSRTC	Initial FNAB, *N* (%)	Final FNAB, *N* (%)	Patients with SFU, *N* (%)	Gender, F:M	SFU/FNAB [the first] (%)	Number of thyroid nodules with SFU (*n* = 221)			ROM			
						Benign tumors, BT	Low-risk neoplasms, LRN	Malignant neoplasms, MN	MN/nodules with SFU (%)	MN/patients with SFU (%)	MN/initial FNAB (%)	MN/ final FNAB (%)
I	42 (7.6)	27 (4.9)	6 (2.9)	3:3	15	3	1	2	33.3	33.3	5	7.4
II	303 (54.6)	303 (54.6)	52 (25.2)	40:12	16.8	39	7	6	11.5	11.5	2	2
III	116 (20.9)	121 (21.8)	60 (29.1)	46:14	51.7	37	12	14	22.2	23.3	12.1	11.6
IV	23 (4.1)	26 (4.7)	17 (8.3)	12:5	73.9	17	3	1	4.8	5.9	4.3	3.8
V	42 (7.6)	43 (7.7)	40 (19.4)	32:8	95.2	6	1	38	84.4	95	90.5	88.4
VI	31 (5.6)	35 (6.3)	31 (15)	24:7	100	-	1	30	96.8	96.8	96.8	85.7
Total	555 (100)	555 (100)	206	157:49	36.9	102	25	91	41.4	44.2	16.4	16.4

Repeated biopsies were often necessitated by non-diagnostic results, fibrosis leading to inadequate cellular samples, or nodule enlargement. New lesions or discrepancies between ultrasound findings (based on the TIRADS scale) and TBSRTC classifications also prompted repeat FNABs, particularly when the surgeon was uncertain about the appropriate extent of surgery (e.g., lobectomy versus total thyroidectomy). In general, biopsies categorized as Bethesda V and VI were not repeated, and total thyroidectomy was performed immediately in these cases.

### Surgery without TBSRTC categorization

In our database, 217 patients underwent thyroid surgery (female-to-male ratio = 160:57, average age = 13.3 ± 3.0 years, range = 3–18 years). Among these, 11 patients were not classified according to the TBSRTC because they had not undergone FNAB.

Of these 11 patients, five were diagnosed with medullary thyroid carcinoma (MTC) (female: male ratio = 3:2), and six had papillary thyroid carcinoma (PTC) in lymph nodes following thyroidectomy (female-to-male ratio = 3:3, average age = 12 years, range = 7–15 years). The reason for surgery in MTC cases was due to a diagnosis of multiple endocrine neoplasia type 2 (MEN2a) with a RET634 mutation (*n* = 3; two 3-year-old patients and one 14-year-old, all with bilateral involvement), MEN2b (*n* = 1; 4-year-old, right lobe involvement), and one case of palliative resection (*n* = 1; 12-year-old). The palliative surgery included resection of the thymus, muscles, and nerves, which were all infiltrated by the tumor. One girl with MEN2a had previously undergone surgery for pheochromocytoma of the adrenal gland.

The diameters of MTC foci in non-palliative cases were 2.2 mm, 1.7 mm, 6 mm, and 25 mm (with elevated calcitonin in the latter patient). In the palliative case, the tumor measured 35 mm and had metastasized to the lungs, caused vocal cord dysfunction, and exerted pressure on the trachea. The only patient with MTC who underwent FNAB was a 14-year-old girl. A 30 mm nodule was detected in the right lobe, and smaller nodules up to 1.2 mm were found in the left lobe, categorized as Bethesda V.

Among the six PTC patients who did not have FNAB, five underwent lateral lymph node dissection and were found to have metastases. One patient had psammoma bodies but no identifiable cancer cells. After a second pathology consultation, this case was interpreted as metastatic disease. The average age of the group with metastatic nodules (*n* = 6) was 12 years (range = 7–15 years).

### Surgery with TBSRTC categorization and histological examination

A total of 206 patients underwent surgery with histological examination as part of their surgical follow-up (SFU). The average age of these patients was 13.4 years (± 2.8), with a range of 3 to 18 years.

#### Bethesda category I (non-diagnostic, ND)

In the Bethesda I (non-diagnostic) category (*n* = 6), two AIT patients were diagnosed with papillary thyroid microcarcinoma (microPTC), one of which was the classical subtype (4.7 mm) and the other the follicular subtype (with nodules measuring 4.7 mm and 5 mm, respectively). One non-AIT patient was diagnosed with non-invasive follicular thyroid neoplasm with papillary-like nuclear features (NIFTP), with a nodule size of 10 mm. The remaining three non-AIT patients were diagnosed with follicular nodular disease (FND). The sizes of the FND nodules were 5 mm and 6 mm for microvesicular patterns and 35 mm for a colloid nodule. Detailed histological findings categorized by the TBSRTC are presented in Table 2, and the differences in the ROM between non-AIT and AIT patients are outlined in Table 3.

**Table 2:** Distribution of SFU findings in thyroid nodules based on histological subtypes and TBSRTC categories in fine-needle aspiration biopsy (FNAB) prior to surgical decision-making. Explanation of calculations: If multiple pathological subtypes or nodules were detected in a single patient, the patient was counted more than once. However, if a multifocal tumor of a single histological type (e.g., multifocal papillary thyroid carcinoma) was present, it was counted as a single tumor in the table. Abbreviations: *FND* follicular nodular disease, *FA* follicular adenoma (currently with follicular adenoma with papillary architecture), *OA* oncocytic adenoma (formerly Hürtle-cell adenoma), *NIFTP* non-invasive follicular thyroid neoplasm with papillary-like nuclear features, *FT-UMP* follicular tumor of uncertain malignant potential, *WDT-UMP* well-differentiated tumor of uncertain malignant potential, *PTC* papillary carcinoma, *FTC* follicular carcinoma, *OC & MTC* oncocytic and medullary carcinoma, *ROM* risk of malignancy, *SFU* surgical follow-up

Category of TBSRTC (final FNAB)	Benign tumors, BT						Low-risk neoplasms, LRN								Malignant neoplasms, MN									
	FND		FA		OA		NIFTP		FT-UMP		WDT-UMP		Hyalini-zing TT		PTC, classic		PTC, follicular		PTC, other subtypes		FTC		OC & MTC	
**AIT**	** − **	** + **	** − **	** + **	** − **	** + **	** − **	** + **	** − **	** + **	** − **	** + **	** − **	** + **	** − **	** + **	** − **	** + **	** − **	** + **	** − **	** + **	** − **	** + **
I	3	-	-	-	-	-	-	1	-	-	-	-	-	-	-	1	-	1	-	-	-	-	-	-
II	29	8	2	-	-		3	-	-	-	2	-	2	-	0	2	2	-	1	-	1	-	-	-
III	23	5	5	1	3	-	2	2	2	1	5	-	-	-	-	-	2	4	3	1	4	-	-	-
IV	9	2	1	-	4	1	1	-	1	1	-	-	-	-	-	-	-	-	-	1	-	-	-	-
V	3	-	1	2	-	-	1	-	-	-	-	-	-	-	9	5	5	2	11	3	1	-	2	-
VI	-	-	-	-	-	-	-	1	-	-	-	-	-	-	10	3	-	-	14	3	-	-	-	-
**Total**	67	15	9	3	7	1	7	4	2	2	7	-	2	-	19	11	9	7	29	8	6	-	2	-

**Table 3:** The TBSRTC outcomes in groups of AIT and non-AIT patients as a prediction in surgical sample

Categories of TBSRTC	Final FNAB, *N*		Patients with SFU, *N*		Gender, F:M		Average nodule size (mm)		Nodules with SFU						ROM					
									Benign tumors, BT		Low-risk neoplasms, LRN		Malignant neoplasms, MN		MN/nodules with SFU (%)		MN/patients with SFU (%)		MN/final FNA (%)	
**AIT**	** − **	** + **	** − **	** + **	** − **	** + **	** − **	** + **	** − **	** + **	** − **	** + **	** − **	** + **	** − **	** + **	** − **	** + **	** − **	** + **
I	22	5	4	2	1:3	2:0	14.0	4.9	3	-	1	-	-	2	0	100	0	100	0	40
II	199	104	40	12	29:11	11:1	21.3	20.3	31	8	7	-	4	2	9.5	20	10	16.6	2	1.9
III	82	39	46	14	36:11	11:3	18.6	12.6	29	6	9	3	9	5	19.1	35.7	19.1	38.5	11	12.8
IV	21	5	13	4	9:4	3:1	12.2	8.3	14	3	2	1	-	1	0	20	0	25	0	20
V	31	12	30	10	23:7	9:1	13.7	9.2	4	2	1	-	28	10	84.8	83.3	93.3	100	90.3	83.3
VI	26	9	24	7	18:6	6:1	19.6	16.6	-	-	-	1	24	6	100	85.7	100	85.7	92.3	66.7
**Total**	381	174	158	48	116:42	41:7	17.9	12.8	81	19	19	5	65	26	39.4	48.9	41.1	54.2	17.1	14.9

#### Bethesda category II (benign, B)

A total of 52 patients were categorized as Bethesda II, indicating benign lesions. The most commonly observed condition was follicular nodular disease (FND), which was diagnosed in 33 patients, 18 of whom had multiple nodules (≥ 2). Among the FND cases, six patients had additional findings: one had follicular adenoma (FA), two had microPTC, two had NIFTP, and one patient had both PTC and WDT-UMP alongside FND. Of the 33 FND cases, six exhibited partial oncocytic changes, with two of these cases occurring in patients with autoimmune thyroiditis (AIT). See SI Fig. 1A for an example of FND with oncocytic changes.

##### 40 Non-AIT patients

The non-AIT subgroup of Bethesda II included 40 patients, with the following diagnoses: two had congenital changes of the thyroglossal duct, 29 had FND (10 of these had multiple nodules), two had FA, three had NIFTP (two of which occurred alongside multiple FND goiter, and one as a singular NIFTP), one had WDT-UMP, one had hyalinizing trabecular tumor (HTT), and one patient had a combination of three different changes (HTT, WDT-UMP, and FND). Interestingly, Actinomyces colonies were found in one case involving a congenital anomaly of the thyroglossal duct. A notable case involved a patient with multiple types of nodules: a 13 mm hyalinizing trabecular tumor (HTT) in the right lobe, a 10 mm follicular adenoma (FA) in the left lobe, and multiple hyperplastic nodules (FND). This patient was initially suspected to have focal Bethesda III. Cytology for low-risk neoplasms is shown in SI Figs. 2 A-D.

Three patients had PTC, and one patient had follicular thyroid carcinoma (FTC) measuring 50 mm in diameter. One case involved a patient with encapsulated follicular PTC (11 mm) who also had a classical microPTC lesion (0.75 mm). Another patient with follicular PTC had two malignant nodules—one measuring 38 mm in the right lobe and the other 10 mm in the left. Another patient with a 3 mm follicular PTC also had an FND nodule.

In the non-AIT FND group with a single nodule, two patients had incidental findings of microPTC. One non-AIT patient with multiple FND (with the largest nodule measuring up to 54 mm in diameter) also had a 3 mm follicular variant of PTC microcarcinoma discovered.

##### 12 AIT patients

In the Benign Lesions group, 12 patients had AIT. Among these, two had microPTCs (both classical variants, measuring 6 mm and 3.5 mm, respectively), six had FND (two with multiple nodules), and four had Graves’ disease, requiring surgery due to thyrotoxicosis. The underdiagnosis of microcarcinoma may be related to the fact that these small lesions were not biopsied during FNAB or missed on ultrasound scans.

#### Bethesda category III (atypia of undetermined significance, AUS)

Bethesda category III (AUS) included 60 patients, with pathology reports showing a variety of outcomes: three patients had fibrotic areas associated with autoimmune thyroiditis, 34 had benign neoplasms, nine had low-risk neoplasms, and 14 were diagnosed with malignant neoplasms. Among the 32 patients with follicular nodular disease (FND), seven had focal oxyphilic changes, and seven had multiple nodules (two of whom also had papillary thyroid carcinoma, PTC).

##### 46 Non-AIT patients

Thirty patients had benign outcomes, including 23 FND (five with multiple nodules), five follicular adenomas (FA), and three oncocytic adenomas (OA) (two unifocal, and one patient with three OA).

Nine patients had low-risk neoplasms, including four cases of well-differentiated tumor of uncertain malignant potential (WDT-UMP), two cases of follicular tumor of uncertain malignant potential (FT-UMP; 6 mm and 8 mm), and one 3 mm NIFTP.

Malignancies were found in nine patients, including four cases of follicular thyroid carcinoma (FTC), one of whom had a 25 mm FTC in the right lobe and an additional 12 mm NIFTP in the left lobe. Five patients had PTC (three follicular subtype, one diffuse sclerosing subtype, and one patient with both an 0.8 mm microPTC solid subtype and a 60 mm WDT-UMP nodule).

##### 14 AIT patients

Three patients had changes related to Hashimoto’s Disease. Five patients had singular FND, and one had FA.

Two patients had NIFTP and one had FT-UMP. Five patients had PTC.

One patient with the follicular subtype of PTC had a 1 mm nodule in the left lobe and a 13 mm NIFTP in the right lobe, along with several FND nodules throughout the gland. Another patient with a 12 mm follicular subtype of PTC also had multiple FND nodules.

One patient had four foci of PTC: two in the left lobe (classical 10 mm and follicular 1.5 mm) and two in the right lobe (classical 1.8 mm and follicular 2.2 mm). Two patients had unifocal PTC of the follicular subtype.

#### Bethesda category IV (follicular neoplasms, FN)

Bethesda category IV lesions, indicative of follicular neoplasms, were diagnosed in 17 patients. Among these, only one patient was found to have a malignant neoplasm.

##### 13 Non-AIT patients

Six patients had FND (one with oxyphilic metaplasia and four with multifocal FND), four had oncocytic adenoma (OA; one with additional hyperplastic nodules, and two OA were partially necrotic), one had FA, and one had NIFTP with FND. One patient had both FT-UMP with oxyphilic metaplasia and FND, classified as Bethesda categories IV and III, respectively (patients were categorized based on the highest Bethesda score).

In two patients with FND, multiple nodules were observed, and pathology revealed the presence of paraganglionic bodies or ultimobranchial rests within the thyroid gland. Exemplary cytology for differential diagnosis between OA, adenomatous colloid nodules (AC), and FTC is shown in SI (Figs. 1 B-D).

##### Four AIT patients

Two patients had unifocal FND lesions, one of which also exhibited oxyphilic metaplasia. One patient had FT-UMP with oxyphilic metaplasia (10 mm). One patient had a 7 mm classical microPTC.

#### Bethesda category V (suspicious for malignancy, SM)

FNAB results in 40 patients (30 non-AIT and 10 AIT) were classified as Bethesda category V (suspicious for malignancy). The histopathological diagnoses within this group included two cases of NIFTP and three rare carcinomas—follicular thyroid carcinoma (FTC), oncocytic carcinoma (OC), and MTC—while the remaining cases were diagnosed as PTC. Of the 32 PTC cases, subtypes included 14 classical, seven follicular, two diffuse sclerosing, and nine mixed types (comprising follicular, classical, and solid subtypes). Additionally, rare variants were noted, including one cribriform with morules, one with focal squamous metaplasia, and one exclusively solid and trabecular. Further details on these subtypes are presented in Supplementary Figs. 3A-D. The size of PTC nodules in histology ranged from 0.3 to 23 mm, with an average nodule size of 13.7 mm in non-AIT patients and 9.2 mm in AIT patients. Seventeen patients presented with multiple PTC nodules, and nine cases were bilateral.

##### 30 Non-AIT patients

In the non-AIT group (*n* = 30), two patients had follicular nodular disease (FND), one of which also exhibited oxyphilic metaplasia, while another had both NIFTP and multiple FND lesions ranging from 12 to 35 mm. In this case, Bethesda categories V and III were applied. One patient had a 32 mm follicular adenoma (FA), another had a 15 mm oncocytic carcinoma (formerly Hürthle cell carcinoma), one had a 30 mm MTC in a 14-year-old female, and one had a 33 mm FTC. The remaining 23 patients had PTC.

##### 10 AIT patients

Among the 10 AIT patients, nine had PTC, and one had both a 5 mm FA nodule and a 0.3 mm microPTC. Additionally, one patient with a 23 mm classical PTC also had a 14 mm FA. These cases were categorized as Bethesda V and III, respectively. As per protocol, patients were assigned to the highest applicable Bethesda category based on their findings.

#### Bethesda category VI (malignant, M)

Among 31 patients classified as Bethesda category VI (malignant), one patient with AIT had a 6 mm NIFTP in addition to a lymphoepithelial cyst. The remaining 30 patients were diagnosed with PTC. Of these, six patients had AIT. Multiple PTC lesions were observed in 22 patients, three of whom had AIT. Twelve cases involved bilateral PTC. Histological subtypes included 13 classical PTCs (three in AIT patients), one columnar variant, and 16 mixed variants (comprising classical, follicular, solid, insular, and tall-cell subtypes), with three of the mixed cases occurring in AIT patients. The size of PTC lesions varied significantly, with extremely small foci (< 5 mm) identified in three patients (0.7 mm, 1.4 mm, and 4 mm), all of whom had multiple lesions. In contrast, four patients exhibited large lesions (> 40 mm), with the largest measuring 52 mm, 54 mm, and two measuring 60 mm.

### TBSRTC performance in postoperative patients

FNAB with subsequent cytological evaluation is a critical tool in surgical decision-making for pediatric patients. In cases of atypia of undetermined significance (AUS) and follicular neoplasm (FN), lobectomy is recommended, while patients with suspicious malignancy (SM) or confirmed malignancy (M) typically undergo total thyroidectomy. Other neoplasms, excluding PTC and MTC, are also treated with lobectomy. We assessed the performance of the Bethesda System for Reporting Thyroid Cytopathology (TBSRTC) based on final FNAB outcomes in patients with solitary functioning nodules (SFN) for the following: (a) PTC, (b) malignancy of any type, and (c) low-risk versus malignant neoplasms, across various TBSRTC categories (see Table 4).

**Table 4:** TBSRCT performance in patients with SFU. Abbreviations: *Se* sensitivity, *Sp* specificity, *PPV* positive predictive value, *NPV* negative predictive value, *Acc* Accuracy

Gold standard and TBSRCT	Group	Sensitivity (%)	Specificity (%)	PPV (%)	NPV (%)	Accuracy (%)
PTC in categories V and VI Bethesda	Non-AIT	86	95	90.7	92.3	91.8
	AIT	61.5	95.5	94.1	67.7	77.1
	Total	78.3	95.1	91.5	86.7	88.3
Malignancies in categories IV, V, and VI Bethesda	Non-AIT	80	83.9	77.6	85.7	82.3
	AIT	65.4	81.8	81	66.7	72.9
	Total	75.8	83.5	78.4	81.4	80.1
Low-risk and malignant neoplasm in categories III, IV, V, and VI Bethesda	Non-AIT	85.7	43.2	63.2	72.7	65.8
	AIT	87.1	58.8	79.4	71.4	72.5
	Total	86.1	46.1	66.9	72.4	68.4

A proportion test revealed *Z*-values of 0.57, 1.32, 1.46, and 1.08 for Bethesda categories II, III, V, and VI, respectively, indicating a lack of statistical significance at the 0.05 level (the critical *Z*-value was 1.6). The proportion test could not be performed for non-AIT patients in Bethesda categories I and IV, as no malignancies were identified in these groups. *P*-values for Bethesda categories II, III, V, and VI were 0.526, 0.145, 0.402, and 0.060, respectively, suggesting no statistically significant differences at the 0.05 threshold.

## Discussion

The increasing accessibility of ultrasound equipment in endocrinological and surgical clinics, along with the rising adoption of point-of-care ultrasonography, has enabled the detection of small thyroid lesions. However, fine needle aspiration biopsy (FNAB) in small lesions carries a risk of false-negative results, as demonstrated in this study. The primary objective of comprehensive thyroid screening is to identify malignancies at an early stage, ideally before metastases occur, thereby reducing the need for aggressive treatments.

Nodules classified as Bethesda categories I and II generally did not require surgery but instead were monitored through follow-up and biopsy if deemed suspicious (e.g., small, asymptomatic nodules with a low risk of malignancy on ultrasound). In our study, eight out of 58 patients (14%) from Bethesda categories I and II who underwent surgery had undetected malignancies. Among these, seven patients (12%) had papillary thyroid carcinoma (PTC) that required an extension of their surgical treatment. Metastases are more commonly observed in children and tend to be associated with larger lesions, although exceptions do exist. In a previous study, the average sizes of non-metastatic and metastatic PTC were 11 mm and 20 mm, respectively [3]. In the current study, two patients in the I group had undetected PTCs measuring 5 mm and 4.7 mm, respectively. In the II group, one patient had an undetected follicular thyroid carcinoma (FTC) measuring 50 mm, and five patients had undetected PTCs. Of these five, two were AIT patients with PTC diameters of 6 mm and 3.5 mm, and three were non-AIT patients with PTC diameters ranging from 3 to 38 mm (including foci of 0.75 mm, 12 mm, and 10 mm). The diameters of the undetected cancers in Bethesda categories I and II suggest that only two of the eight malignant nodules had the potential for metastasis based on size alone. However, histopathological examination revealed metastases in four of these patients, with the smallest metastatic nodule measuring 5 mm. This underscores the challenges of relying solely on FNAB for small lesions and highlights the importance of vigilant surgical and histological follow-up in cases where malignancy risk remains uncertain.

Patients with autoimmune thyroiditis (AIT) and papillary thyroid carcinoma (PTC) at initial presentation were reported to have lesions of similar size to those in non-AIT patients, although these lesion sizes were larger than in AIT patients whose PTC was detected in late follow-up [2]. In this study, the prevalence of AIT among patients with thyroid nodules was 23.3% (48/206), consistent with findings in existing literature [13]. Additionally, The Bethesda System for Reporting Thyroid Cytopathology (TBSRTC) demonstrated comparable diagnostic accuracy across nodules of varying sizes, including those ≤ 0.5 cm, > 0.5–1 cm, > 1–2 cm, and > 2–4 cm in diameter [19]. Notably, herein the smallest PTC detected in the “suspicious for malignancy” group (Bethesda category V) measured 3 mm. These findings indicate that both large and small PTCs can be misdiagnosed, potentially due to factors such as missed aspiration during biopsy, lack of visibility on ultrasound, or lack of distinct cytological changes. Our experience indicates that the classical subtype of PTC is more readily detectable than the follicular subtype. Misdiagnosis of larger lesions is particularly concerning, as they are more likely to metastasize.

In the overall cohort, the sensitivity and specificity were 78% and 95% for Bethesda categories V and VI (for PTC) and 86% and 46% for Bethesda categories III to VI (for low-risk and malignant neoplasms), respectively. Sensitivity for detecting PTC in TBSRTC categories V and VI is higher in non-AIT patients (86%) compared to AIT patients (61.5%). The lower sensitivity in AIT patients implies that additional surgery, such as the removal of the second thyroid lobe, may be required more frequently. This discrepancy in sensitivity may be attributed to the presence of fibrosis and reduced cellularity in AIT-affected tissues, as well as inflammation-related fragmentation of follicles, which can mimic the appearance of malignant tissues. The sensitivity FNAB for detecting low-risk and malignant neoplasms in Bethesda categories III to VI was comparable between patients with AIT and those without, at approximately 86% and 87%, respectively. Further, the specificity of FNAB for detection of PTC was high, around 95% in both AIT and non-AIT groups, but the specificity for detecting low-risk and malignant neoplasms was low (43% in non-AIT and 59% in AIT patients). Current treatment guidelines recommend lobectomy without adjunctive chemotherapy or radiotherapy for low-risk neoplasms [12]. However, no statistically significant difference in the risk of malignancy (ROM) was observed between the AIT and non-AIT groups. There was a trend indicating that Bethesda categories III and V may carry a higher risk for patients with AIT.

Data from the literature suggest an increased ROM in Hashimoto’s thyroiditis patients with thyroid nodules, encompassing both intermediate and malignant cytology [20, 21]. Furthermore, thyroid nodules in Hashimoto’s patients are more frequently multiple. Some researchers hypothesize that the elevated risk of PTC in Hashimoto’s thyroiditis may be due to chronic inflammation and elevated thyroid-stimulating hormone (TSH) levels [2]. However, in a previous study, AIT patients without PTC exhibited higher TSH levels compared to all PTC groups, including both AIT and non-AIT PTC patients [3]. This finding challenges the notion that elevated TSH is a major driver of thyroid carcinogenesis. The immunological mechanisms underlying PTC in both pediatric and adult AIT patients require further investigation to better understand these diagnostic challenges [22–24]. The high specificity of FNAB for PTC, combined with its low specificity for low-risk neoplasms, suggests that nearly all patients diagnosed with neoplasms in Bethesda categories V and VI should undergo total thyroidectomy. The specificity of approximately 50% for detecting malignant and low-risk neoplasms (cumulative cases from Bethesda III to VI) in asymptomatic nodules suggests that up to half of these cases may be overtreated. Literature findings report sensitivity and specificity of 69% and 88% for Bethesda V and VI and 90% and 51% for Bethesda III to VI, consistent with our results [25].

Bethesda categories III and IV are not specifically aimed at detecting PTC. However, in pediatric cases, hemithyroidectomy is often performed, with additional surgery on the remaining thyroid lobe or, in some cases, treatment with radioactive iodine (I-131) if PTC is identified upon histopathological examination [12]. Of the 77 patients in this study with Bethesda categories III and IV, seven with category III and one with category IV were diagnosed in histology with PTC, indicating that approximately 10% of pediatric patients may require additional surgery following an initial lobectomy. Low-risk neoplasms proved challenging to diagnose using The Bethesda System for Reporting Thyroid Cytopathology (TBSRTC). Twenty-four patients with low-risk neoplasms were distributed among the following categories: I, 1; II, 7; III, 12; IV, 1; and VI, 1. Tumor architecture, as depicted in TBSRTC, can influence these findings [26–28].

In the Bethesda category V, 4 out of 40 patients (10%) were overtreated, while 1 out of 31 patients (3%) in the Bethesda category VI was overtreated. The challenge in distinguishing between Bethesda category III and IV may be due to the percentage of oxyphilic cells present. For a diagnosis of oncocytic adenoma (OA) under Bethesda category IV, more than 75% of the nodule must consist of oxyphilic cells, whereas a lower percentage is classified as follicular adenoma (FA) under Bethesda category III [29]. Oncocytic metaplasia can occur in hyperplastic nodules, often in association with Hashimoto’s thyroiditis, as well as in OA and oncocytic carcinoma (OC) [29].

Vuong et al. (2021) reported the frequency of nodules classified as Bethesda I, II, III, IV, and VI as follows: 11.4%, 54.7%, 10.3%, 8.4% (range 6.1–16.6%), 3.3%, and 11.7%, respectively [30]. In our cohort, we observed a lower frequency of categories I, II, and IV, conversely, a higher frequency of Bethesda III, V, and VI, with rates of 2.9%, 25.2%, 29.1%, 8.3%, 19.4%, and 15% for Bethesda categories from I to VI, respectively.

Ali et al. (2023) reported the risk of malignancy (ROM) values for each Bethesda category as follows: 14% (range 0–33%) for I, 6% (range 0–27%) for II, 28% (range 11–54%) for III, 50% (range 28–100%) for IV, 81% (range 40–100%) for V, and 98% (range 86–100%) for VI [7]. In this study, the ROM in all biopsied nodules, aside from category IV of Bethesda, was consistent with reported values in the literature across TBSRTC categories: 33.3%, 11.5%, 22.2%, 4.8%, 84.4%, and 96.8% for Bethesda categories I to VI, respectively.

In patients, who had thyroid surgery, the ROM was higher in I, II, III, and IV categories for patients with AIT compared to non-AIT, while ROM was lower in Bethesda category VI. This variation may be attributed to the reported performance of FNAB on nodules ≥ 1.0 cm in diameter in many studies, whereas our center performs FNAB on nodules ≥ 0.4 cm. Another factor contributing to this difference is our center’s protocol of conducting microscopic examination of the entire thyroid surgical sample, irrespective of the Bethesda category. In contrast, other centers typically perform full histological examination only for Bethesda V and VI categories. That may result in a higher detection rate of microcarcinomas in our center.

## Conclusions

FNAB using The Bethesda System for Reporting Thyroid Cytopathology (TBSRTC) can detect malignancies ≥ 3 mm in diameter. In our study, FNAB with Bethesda categories I and II, combined, missed approximately 12% of papillary thyroid carcinoma cases. About 10% of patients with Bethesda categories III and IV required reoperation of the contralateral thyroid lobe. Additionally, 10% and 3% of patients categorized as Bethesda V and VI, respectively, were overtreated. TBSRTC is an effective method for assessing thyroid cytology and is valuable in managing pediatric thyroid nodules. The sensitivity for detecting malignancy across categories Bethesda III–VI was approximately 86% in both AIT and non-AIT patients. However, the sensitivity for papillary thyroid carcinoma detection, calculated in Bethesda categories V and VI, was 86% in non-AIT patients and 61.5% in AIT patients. These findings underscore the importance of considering surgical intervention in pediatric patients with Bethesda III-VI cytology, particularly in those with AIT.

## Supplementary information

Below is the link to the electronic supplementary material.

Supplementary file1 (DOCX 1783 KB)

## Data Availability

There is no consent to share non-anonymized patients’ data (according to the approval of The Committee for Scientific Research Ethics of the Jagiellonian University Medical College, opinion number: 118.0043.1.103.2024).

## References

[1] Niedziela M (2006) Pathogenesis, diagnosis and management of thyroid nodules in children. Endocr Relat Cancer 13(2):427–453. 10.1677/erc.1.00882 16728572 10.1677/erc.1.00882

[2] Januś D, Kujdowicz M, Wójcik M et al (2023) Ultrasound evolution of parenchymal changes in the thyroid gland with autoimmune thyroiditis in children prior to the development of papillary thyroid carcinoma – a follow-up study. Front Endocrinol (Lausanne) 14(April):1–13. 10.3389/fendo.2023.1172823 10.3389/fendo.2023.1172823 PMC10130420 37124746

[3] Januś D, Wójcik M, Taczanowska-Niemczuk A et al (2023) Ultrasound, laboratory and histopathological insights in diagnosing papillary thyroid carcinoma in a paediatric population: a single centre follow-up study between 2000–2022. Front Endocrinol (Lausanne) 14(May):1–17. 10.3389/fendo.2023.1170971 10.3389/fendo.2023.1170971 PMC10233204 37274328

[4] Guleria P, Srinivasan R, Rana C, Agarwal S (2022) Molecular landscape of pediatric thyroid cancer: a review. Diagnostics 12(12):1–21. 10.3390/diagnostics12123136 10.3390/diagnostics12123136 PMC9776958 36553142

[5] Jarząb B, Dedecjus M, Lewiński A et al (2022) Diagnostyka i leczenie raka tarczycy u chorych dorosłych — Rekomendacje Polskich Towarzystw Naukowych oraz Narodowej Strategii Onkologicznej. Aktualizacja na rok 2022. Endokrynol Pol. 73(2):173–300. 10.5603/EP.a2022.0028 10.5603/EP.a2022.0028 35593680

[6] Guidelines of the Papanicolaou Society of Cytopathology for the examination of fine-needle aspiration specimens from thyroid nodules (1996) The Papanicolaou society of cytopathology task force on standards of practice. Diagn Cytopathol 15(1):84–89. 10.1002/(SICI)1097-0339(199607)15:1%3c84::AID-DC18%3e3.0.CO;2-8 8807260 10.1002/(SICI)1097-0339(199607)15:1<84::AID-DC18>3.0.CO;2-8

[7] Ali SZ, Baloch ZW, Cochand-Priollet B, Schmitt FC, Vielh P, Vanderlaan PA (2023) The 2023 Bethesda system for reporting thyroid cytopathology. Thyroid 33(9):1039–1044. 10.1089/thy.2023.0141 37427847 10.1089/thy.2023.0141

[8] Hsiao V, Massoud E, Jensen C et al (2022) Diagnostic accuracy of fine-needle biopsy in the detection of thyroid malignancy: a systematic review and meta-analysis. JAMA Surg 157(12):1105–1113. 10.1001/jamasurg.2022.4989 36223097 10.1001/jamasurg.2022.4989 PMC9558056

[9] WHO Classification of Tumours Editorial Board (2022) Endocrine and neuroendocrine tumours. 5th ed. International Agency for Research on Cancer. https://tumourclassification.iarc.who.int/chapters/53

[10] Amin MB, Edge S, Greene F et al (2017) AJCC Cancer Staging Manual, 8th edn. Springer International Publishing

[11] Francis GL, Waguespack SG, Bauer AJ et al (2015) Management guidelines for children with thyroid nodules and differentiated thyroid cancer. Thyroid 25(7):716–759. 10.1089/thy.2014.0460 25900731 10.1089/thy.2014.0460 PMC4854274

[12] Bojarsky M, Baran JA, Halada S et al (2023) Outcomes of ATA low-risk pediatric thyroid cancer patients not treated with radioactive iodine therapy. J Clin Endocrinol Metab 108(12):3338–3344. 10.1210/clinem/dgad322 37265226 10.1210/clinem/dgad322 PMC10655549

[13] Keefe G, Culbreath K, Cherella CE et al (2022) Autoimmune thyroiditis and risk of malignancy in children with thyroid nodules. Thyroid 32(9):1109–1117. 10.1089/thy.2022.0241 35950619 10.1089/thy.2022.0241

[14] Pasala P, Francis GL (2017) Autoimmune thyroid diseases in children. Expert Rev Endocrinol Metab 12(2):129–142. 10.1080/17446651.2017.1300525 30063425 10.1080/17446651.2017.1300525

[15] De Luca F, Santucci S, Corica D, Pitrolo E, Romeo M, Aversa T (2013) Hashimoto’s thyroiditis in childhood: presentation modes and evolution over time. Ital J Pediatr 39:17–19. 10.1186/1824-7288-39-8 23363471 10.1186/1824-7288-39-8 PMC3567976

[16] Ansaldi N, Palmas T, Corrias A et al (2003) Autoimmune thyroid disease and celiac disease in children. J Pediatr Gastroenterol Nutr 37(1):63–66. 10.1097/00005176-200307000-00010 12827007 10.1097/00005176-200307000-00010

[17] Guille JT, Opoku-Boateng A, Thibeault SL, Chen H (2015) Evaluation and management of the solitary thyroid nodule. Oncologist 20:19–27 25480825 10.1634/theoncologist.2014-0115 PMC4294601

[18] Heider A, Arnold S, Jing X (2020) Bethesda system for reporting thyroid cytopathology in pediatric thyroid nodules experience of a tertiary care referral center. Arch Pathol Lab Med 144(4):473–477. 10.5858/arpa.2018-0596-OA 31403334 10.5858/arpa.2018-0596-OA

[19] Koo DH, Song KS, Kwon H et al (2016) (2016) Does tumor size influence the diagnostic accuracy of ultrasound-guided fine-needle aspiration cytology for thyroid nodules? Int J Endocrinol. 1:3803647. 10.1155/2016/3803647 10.1155/2016/3803647 PMC5059591 27774103

[20] De Morais NS, Stuart J, Guan H et al (2019) The impact of Hashimoto thyroiditis on thyroid nodule cytology and risk of thyroid cancer. J Endocr Soc 3(4):791–800. 10.1210/js.2018-00427 30963137 10.1210/js.2018-00427 PMC6446886

[21] Boi F, Pani F, Mariotti S (2017) Thyroid autoimmunity and thyroid cancer: review focused on cytological studies. Eur Thyroid J 6(4):178–186. 10.1159/000468928 28868258 10.1159/000468928 PMC5567004

[22] Dos Santos Valsecchi VA, Betoni FR, Ward LS, Cunha LL (2024) Clinical and molecular impact of concurrent thyroid autoimmune disease and thyroid cancer: from the bench to bedside. Rev Endocr Metab Disord 25(1):5–17. 10.1007/s11154-023-09846-w 37889392 10.1007/s11154-023-09846-w

[23] Chiari D, Pirali B, Perano V, Leone R, Mantovani A, Bottazzi B (2023) The crossroad between autoimmune disorder, tissue remodeling and cancer of the thyroid: the long pentraxin 3 (PTX3). Front Endocrinol (Lausanne) 14(March):1–6. 10.3389/fendo.2023.1146017 10.3389/fendo.2023.1146017 PMC10070760 37025408

[24] Rossi ED, Adeniran AJ, Faquin WC (2019) Pitfalls in thyroid cytopathology. Surg Pathol Clin 12(4):865–881. 10.1016/j.path.2019.08.001 31672295 10.1016/j.path.2019.08.001 PMC7404573

[25] Canberk S, Barroca H, Girão I et al (2022) Performance of the Bethesda system for reporting thyroid cytology in multi-institutional large cohort of pediatric thyroid nodules: a detailed analysis. Diagnostics. 12(1):179. 10.3390/diagnostics12010179 35054346 10.3390/diagnostics12010179 PMC8774335

[26] Strickland KC, Howitt BE, Barletta JA, Cibas ES, Krane JF (2018) Suggesting the cytologic diagnosis of noninvasive follicular thyroid neoplasm with papillary-like nuclear features (NIFTP): A retrospective analysis of atypical and suspicious nodules. Cancer Cytopathol 126(2):86–93. 10.1002/cncy.21922 28914983 10.1002/cncy.21922

[27] Canini V, Leni D, Pincelli AI et al (2019) (2019) Clinical-pathological issues in thyroid pathology: study on the routine application of NIFTP diagnostic criteria. Sci Rep 9(1):1–9. 10.1038/s41598-019-49851-1 31515532 10.1038/s41598-019-49851-1 PMC6742662

[28] Scappaticcio L, Trimboli P, Bellastella G, et al (2023) Prediction of classical versus non classical papillary thyroid carcinoma subtypes from cytology of nodules classified according to TIRADS. Endocrine. Published online 2023. 10.1007/s12020-023-03604-3 10.1007/s12020-023-03604-3 PMC11076311 38001322

[29] Asa SL (2004) My approach to oncocytic tumours of the thyroid. J Clin Pathol 57(3):225–232. 10.1136/jcp.2003.008474 14990587 10.1136/jcp.2003.008474 PMC1770246

[30] Vuong HG, Chung DGB, Ngo LM et al (2021) The use of the Bethesda system for reporting thyroid cytopathology in pediatric thyroid nodules: a meta-analysis. Thyroid. 31(8):1203–1211. 10.1089/thy.2020.0702 33504264 10.1089/thy.2020.0702

