# Supplementary material for: Thyroid cytology in pediatric patients: a single-center study from 2015 to 2023—is there a necessity for distinct treatment approaches for patients with and without autoimmune thyroiditis?
Source: Virchows Arch. 2024 Nov 5;486(6):1175–85. doi: 10.1007/s00428-024-03959-6 (PMC12213851; doi:10.1007/s00428-024-03959-6)
Supplement: Supplementary file 1 — Supplementary file1 (DOCX 1783 KB) [file 428_2024_3959_MOESM1_ESM.docx]

# **Supplemantary Information**


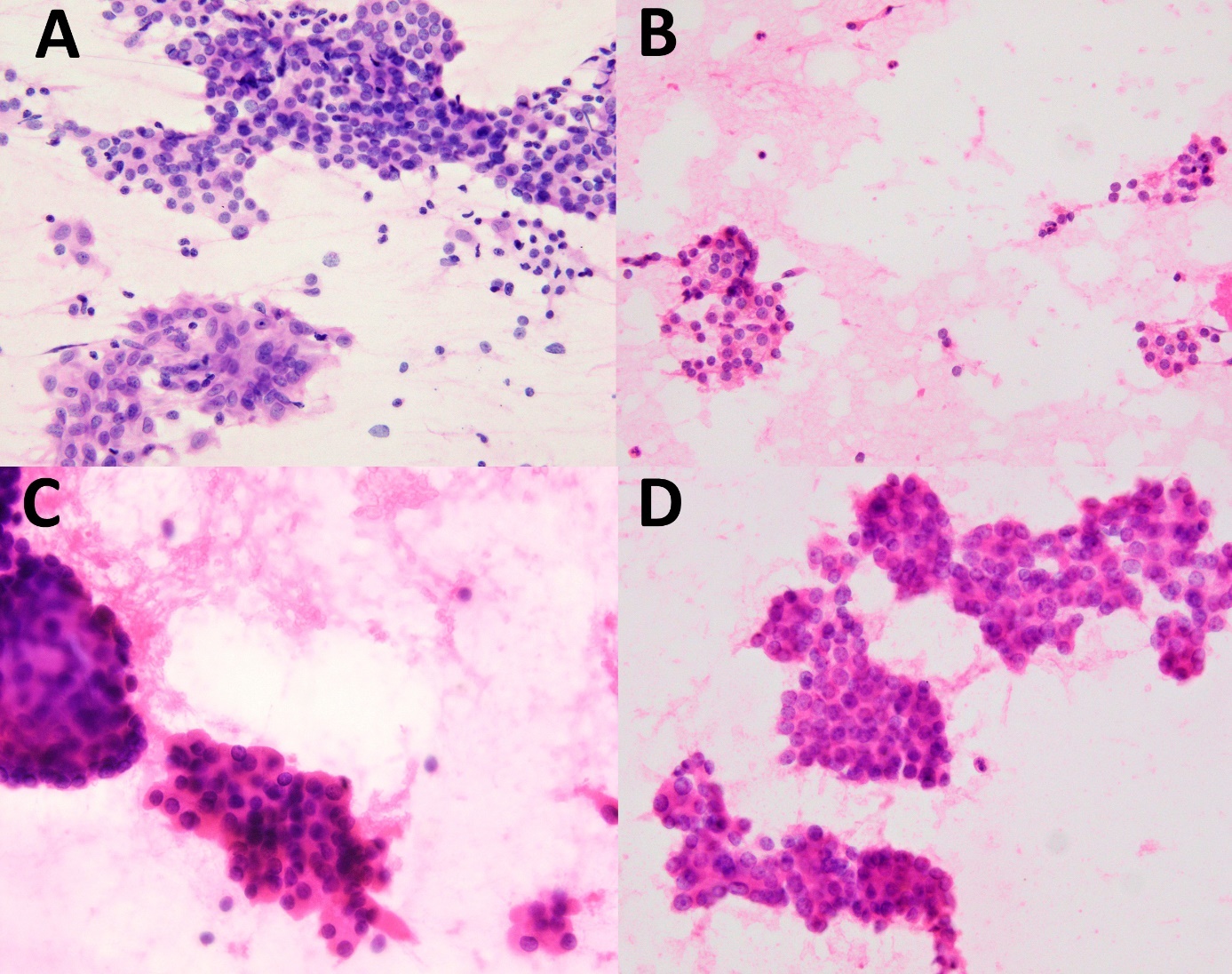


**Figure 1**. Cytological examples of follicular and oxyphilic changes: **A** – FND, **B** – FTC, **C** – OA, **D** – OC, in patients of III (B), IV (A, C) and V (D) TBSRCT. HE; A, B x 200; C, D x400. In FND the large, irregular groups of normotypic thyrocytes prevail in number over the irregular, longitudinal small fascicles of oncocytic cells. FTC shows quite numerous small groups of cells with round, slightly enlarged nuclei. OA consists of groups of cells of various sizes with strongly eosinophilic cytoplasm and some normocytic thyrocytes. OC show numerous irregular groups of thyrocytes of various sizes with eosinophilic cytoplasm and enlarged, hypochromatic nuclei in some cases with prominent nucleoli. In malignancies a few, scattered neutrophils are seen.


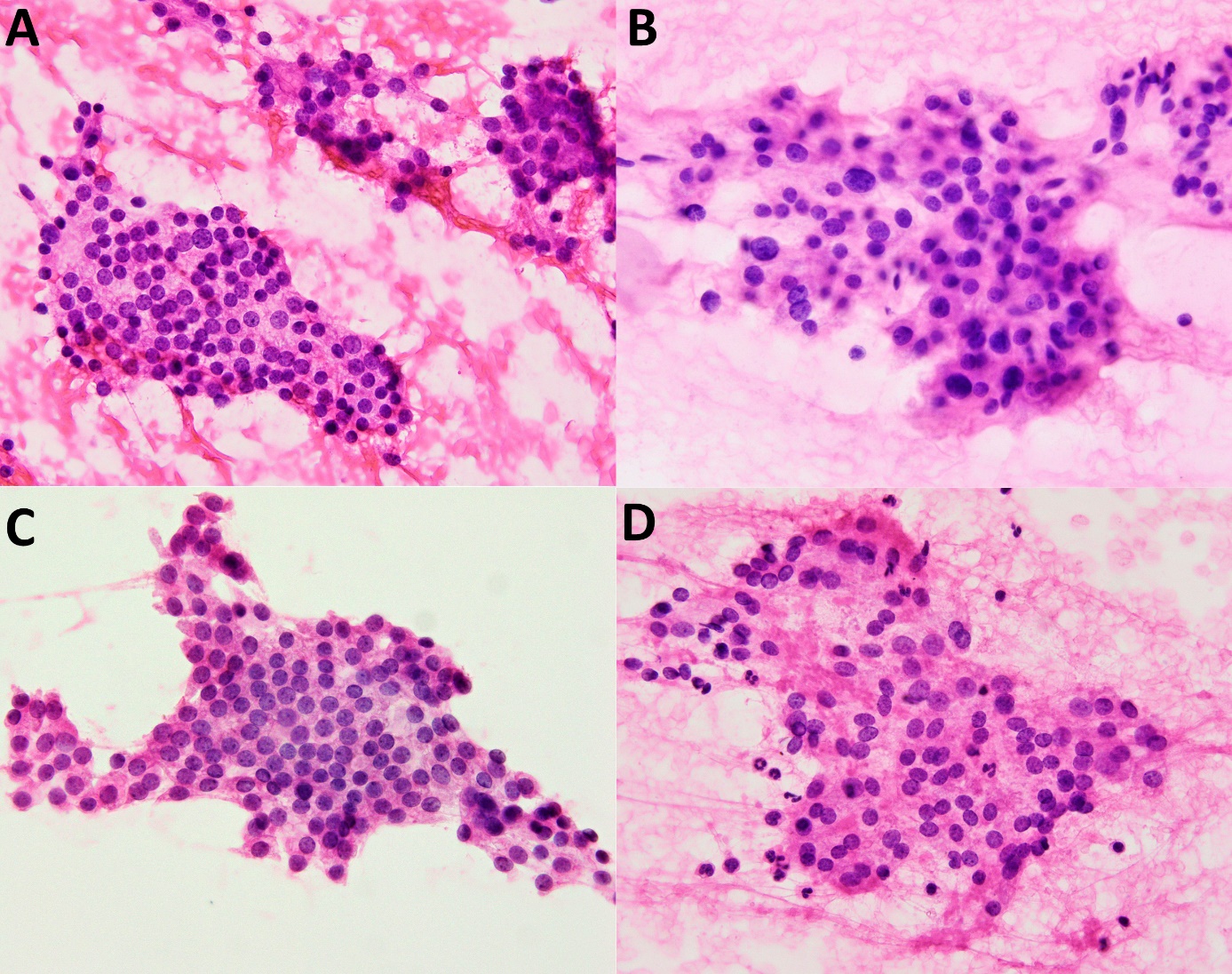


**Figure 2**. Cytological examples of the low-risk neoplasms of Bethesda category III (AUS) are: **A** – NIFTP, **B** – FT-UMP, **C** – WDT-UMP, **D** – Hyalinizing TT. HE, x500. The NIFTP and WDT-UMP consist of irregular groups of thyrocytes of various sizes with features of PTC (enlarged, hypochromatic, and grooved nuclei). The nuclear:cytoplasmic ratio is high. The FT-UMP consists of irregular groups of thyrocytes of various sizes and shapes of nuclei. The hyalinizing TT consist of a very irregular groups of thyrocytes with nuclear features of PTC and a large amount of smashed cytoplasm.


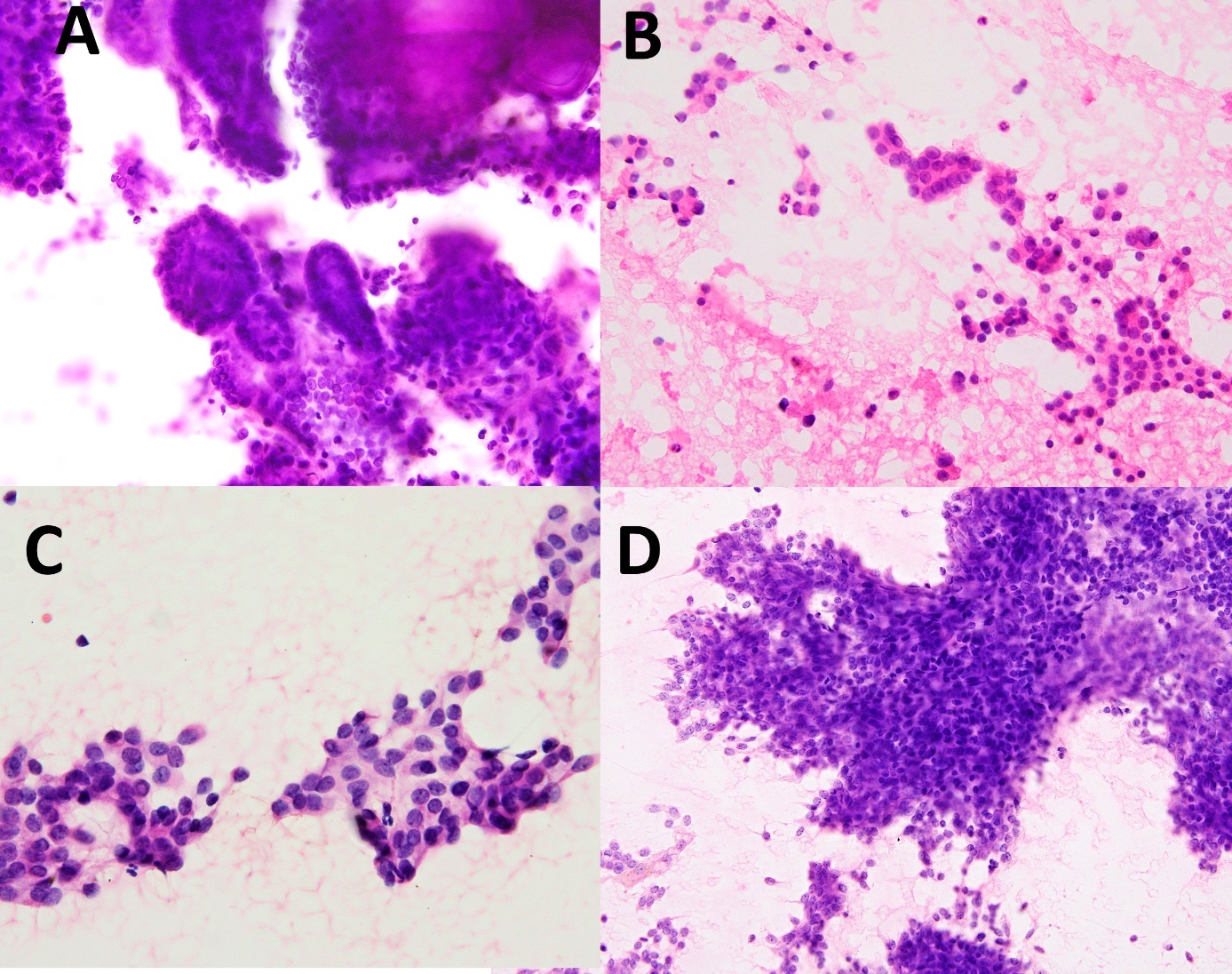


**Figure 3**. Cytological examples of PTC subtypes: **A** – papillary, **B** – follicular, **C** – diffuse sclerosing, **D** – columnar, in V (B, C) and VI (A, D) TBSRCT. HE; A x400, B and D x200, C x500. (**A**) The classic subtype of PTC consists of papillae of thyrocytes with enlarged, hypochromatic nuclei with grooves; the nuclei overlap each other; psammoma bodies can be observed. (**B**) The follicular type of PTC has elusive morphology similar to FTC but the nuclear features of PTC in some ~~of~~ groups are the key to correct diagnosis. (**C**) Cytology of diffuse sclerosing PTC presented with very scant, medium-sized, irregular groups of thyrocytes with enlarged, hypochromatic nuclei. In some of the cells there were grooves and pseudoinclusions. (**D**) The columnar subtype of PTC consists of large, irregular papillae and ~~also~~ scattered medium and small sized groups of cells with PTC features; the edges of papillae are irregular, cells are quite longitudinal and a thin, flag-like cytoplasm is present.
